# Supplementary material for: Kinetic control of tunable multi-state switching in ferroelectric thin films
Source: Nat Commun. 2019 Mar 20;10:1282. doi: 10.1038/s41467-019-09207-9 (PMC6427024; doi:10.1038/s41467-019-09207-9)
Supplement: Supplementary file 1 — Supplementary Information [file 41467_2019_9207_MOESM1_ESM.pdf]

Supplementary Information

Tunable, Multi-state Switching in Ferroelectric Thin Films

R. Xu *et al.*

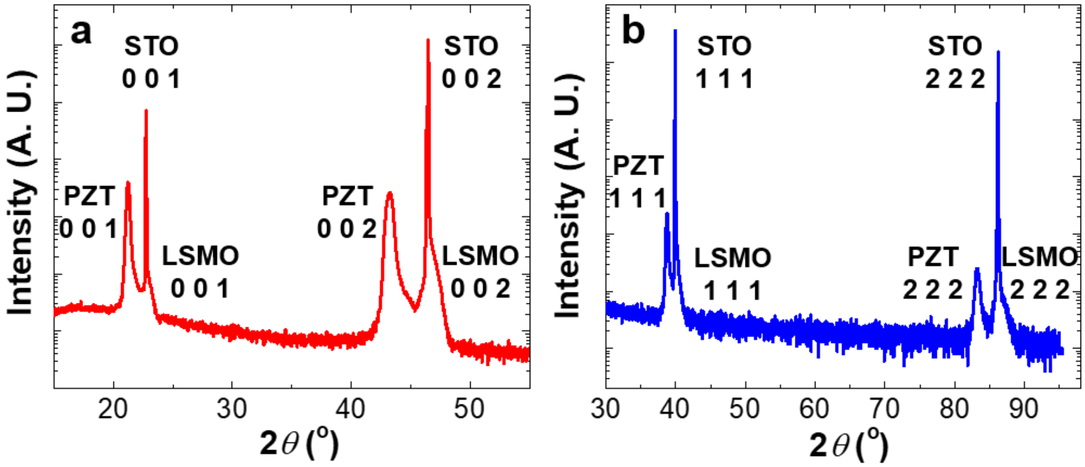

Supplementary Figure 1 | X-ray diffraction analysis. Wide-angle, θ-2θ X-ray diffraction scans for **a**, (001)- and **b**, (111)-oriented $PbZr_{0.2}Ti_{0.8}O_3/La_{0.7}Sr_{0.3}MnO_3/SrTiO_3$ heterostructures. These results reveal that $PbZr_{0.2}Ti_{0.8}O_3$ films are epitaxial and single-phase.

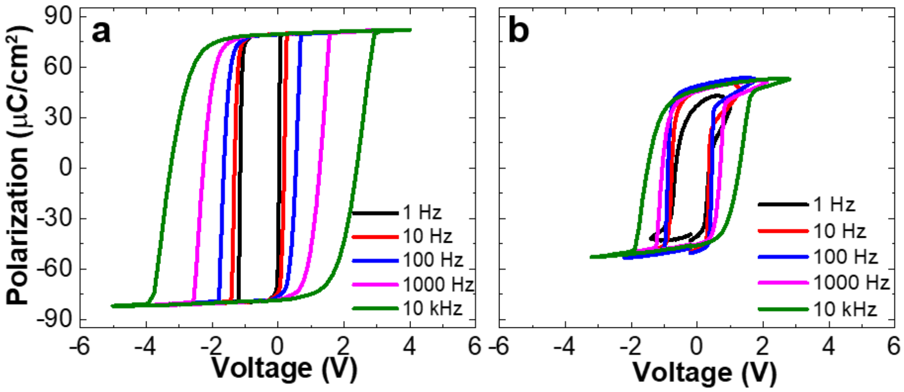

Supplementary Figure 2 | Ferroelectric hysteresis loop measurement. Ferroelectric hysteresis loops measured as a function of frequency for **a**, (001)- and **b**, (111)-oriented $La_{0.7}Sr_{0.3}MnO_3/PbZr_{0.2}Ti_{0.8}O_3/La_{0.7}Sr_{0.3}MnO_3/SrTiO_3$ capacitor structures. These films exhibit symmetric, well-saturated hysteresis loops that are maintained down to at least 1 Hz indicating low leakage and excellent ferroelectric properties.

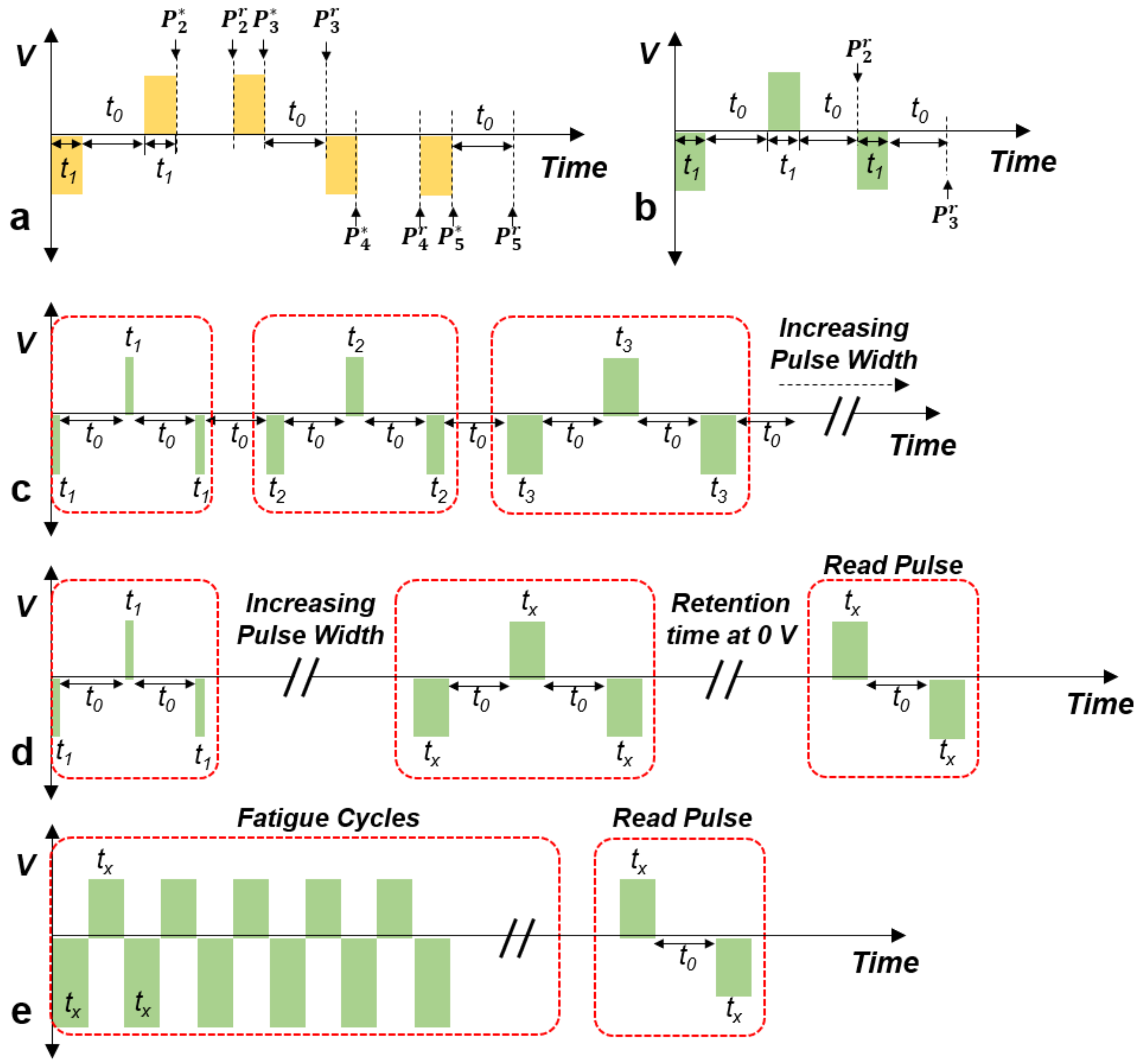

Supplementary Figure 3 | Pulse sequences used in the pulse-switching measurements. a, The pulse sequence used in conventional positive-up-negative-down (PUND) measurements. **b,** The modified PUND pulse sequence used in our measurements. **c,** The modified PUND measured as a function of pulse width for constant pulse voltages. **d,** The pulse sequence used in retention measurement. **e**, The pulse sequence used in fatigue measurement.

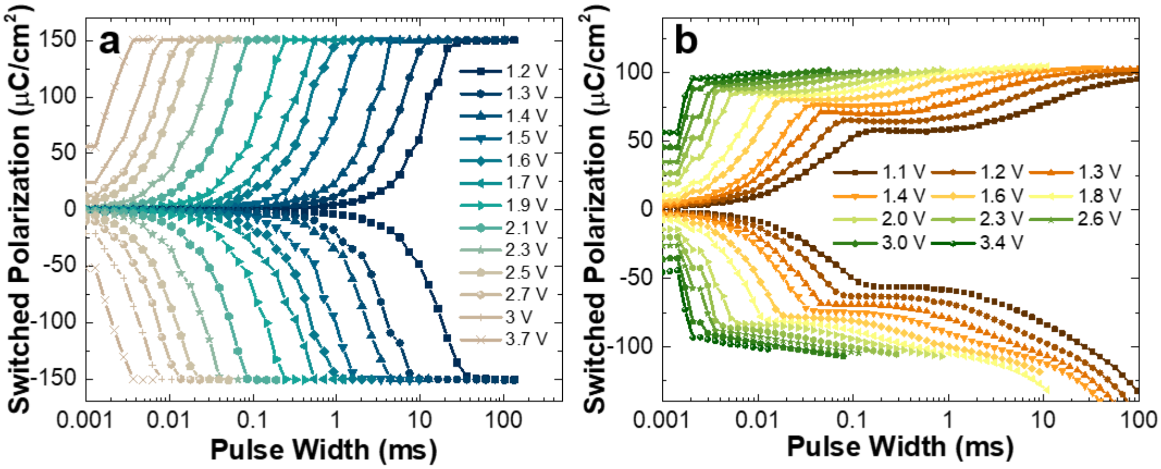

Supplementary Figure 4 | The full data profile of pulse-switching measurements. The switched positive and negative remanent polarization measured as a function of pulse width for different pulse voltages in **a,** (001)- and **b,** (111)-oriented heterostructures.

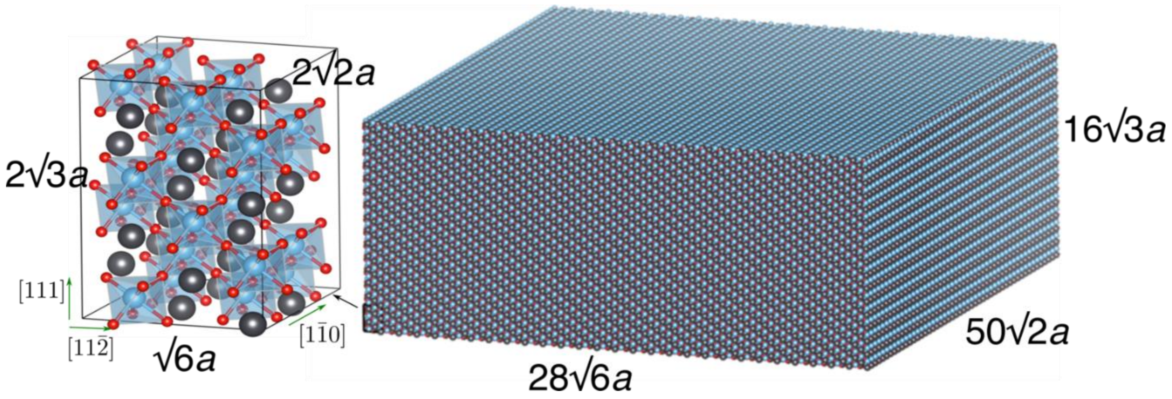

Supplementary Figure 5 | The supercell used in molecular dynamics simulations. The molecular dynamics simulations were performed on a (111)-oriented 28√6×50√2×16√3 supercell of $PbTiO_3$ (672,000 atoms) with periodic boundary conditions (PBCs) and a bond-valence-based interatomic potential parameterized from first-principles. The supercell has the Cartesian axes aligned along crystallographic axes $[11\bar{2}]$, $[1\bar{1}0]$, and [111], respectively.

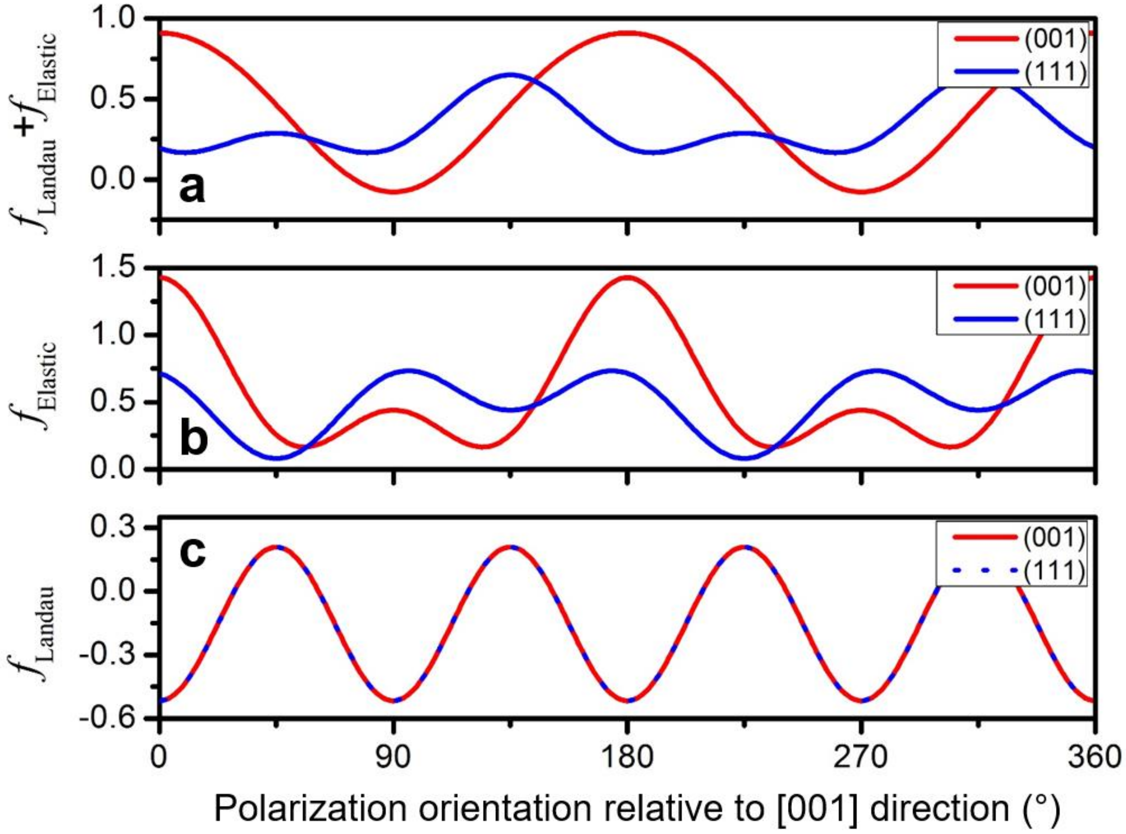

Supplementary Figure 6 | The phase-field energy landscape for polarization switching. a, Total, **b,** elastic, and **c,** Landau energies calculated as a function of polarization orientation. Regardless of film orientation, the Landau energy exhibits local minima at polarization orientations along 0°, 90°, 180°, and 270° due to the tetragonal symmetry of the $PbZr_{0.2}Ti_{0.8}O_3$ films. The elastic energy varies significantly and, therefore, the total energy, which is a sum of the Landau and elastic energies, differs for (001)- and (111)-oriented heterostructures. The angle between the two total energy minima is 180° and ~90° for (001)- and (111)-oriented heterostructures, respectively, which suggests 180° switching is favored in (001)-oriented films, while 90° switching is favored in (111)-oriented films.

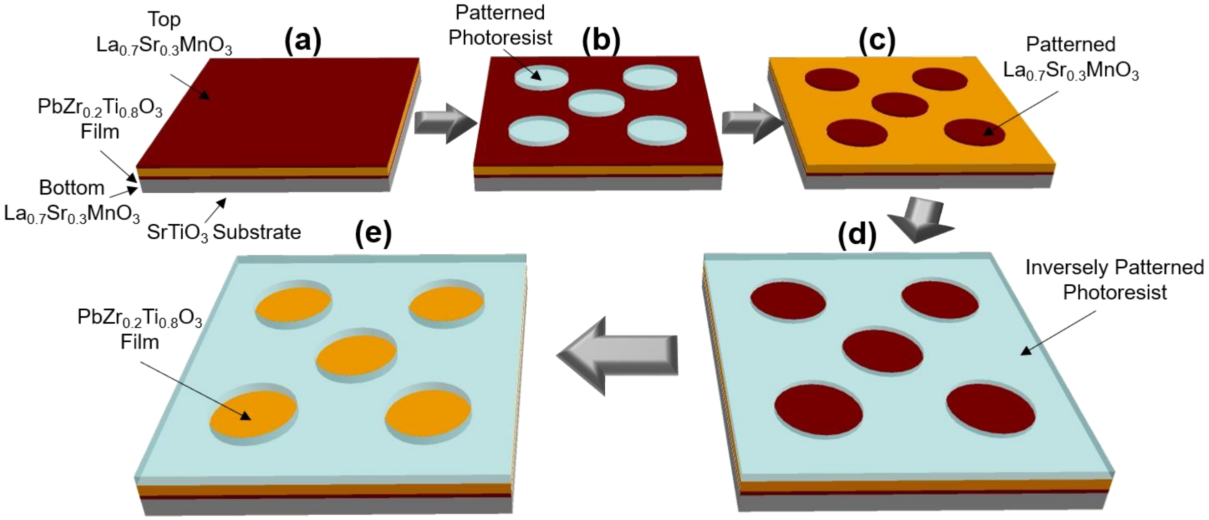

Supplementary Figure 7 | The schematic of the fabrication process to enable domain-structure characterization. a, The as-grown heterostructure before patterning. **b,** A layer of photoresist is patterned onto the as-grown heterostructures using photolithography which covers the circular electrode regions only. **c,** Using a dilute H_3PO_4 acid (dilution ratio 1 part acid : 5 parts water) the uncovered $La_{0.7}Sr_{0.3}MnO_3$ was etched away within 30 seconds leaving circular $La_{0.7}Sr_{0.3}MnO_3$ covered by photoresist. Subsequently, the photoresist was removed by acetone. **d,** Multiple capacitors were pre-poled into different polarization states and then a layer of inversely patterned photoresist was coated by photolithography that only covers the exposed $PbZr_{0.2}Ti_{0.8}O_3$ regions. **e,** The uncovered top $La_{0.7}Sr_{0.3}MnO_3$ electrodes were then etched away using dilute H_3PO_4 acid as noted previously. The inversely-patterned photoresist was left on the film serving as a marker that aids the accurate location and imaging of regions that were under the pre-poled capacitors.

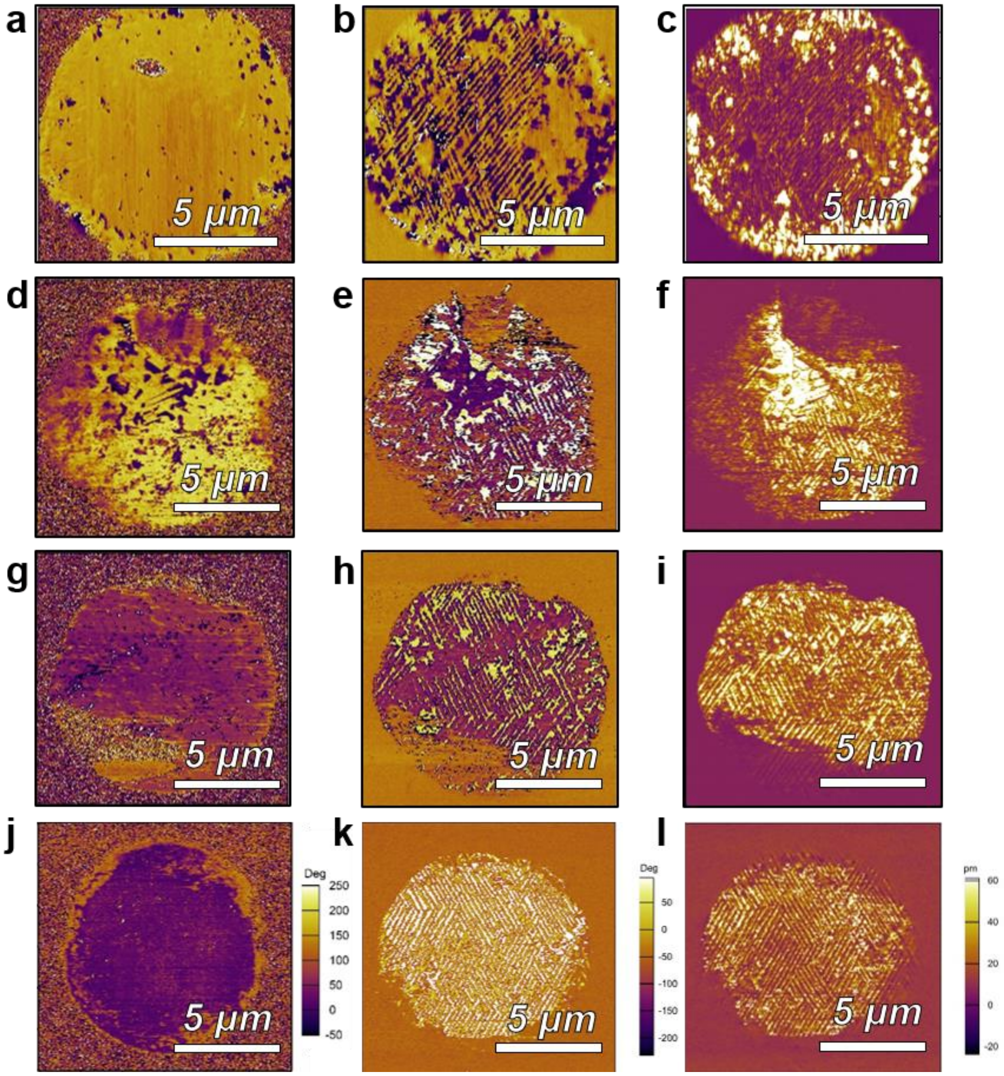

Supplementary Figure 8 | Piezoresponse force microscopy imaging of the domain structures in regions under the pre-poled capacitors. Piezoresponse force microscopy measurements of the domain structures inside the capacitor area for the four pre-poled polarization states discussed in Fig. 3, including those for the **a-c**, fully-up-poled state, **d-f**, the intermediate state poled at 1.1 V, **g-i**, the intermediate state poled at 1.6 V, and **j-l**, the fully-down-poled state. The left column is vertical phase, the center column is lateral phase, and the right column is lateral amplitude images.

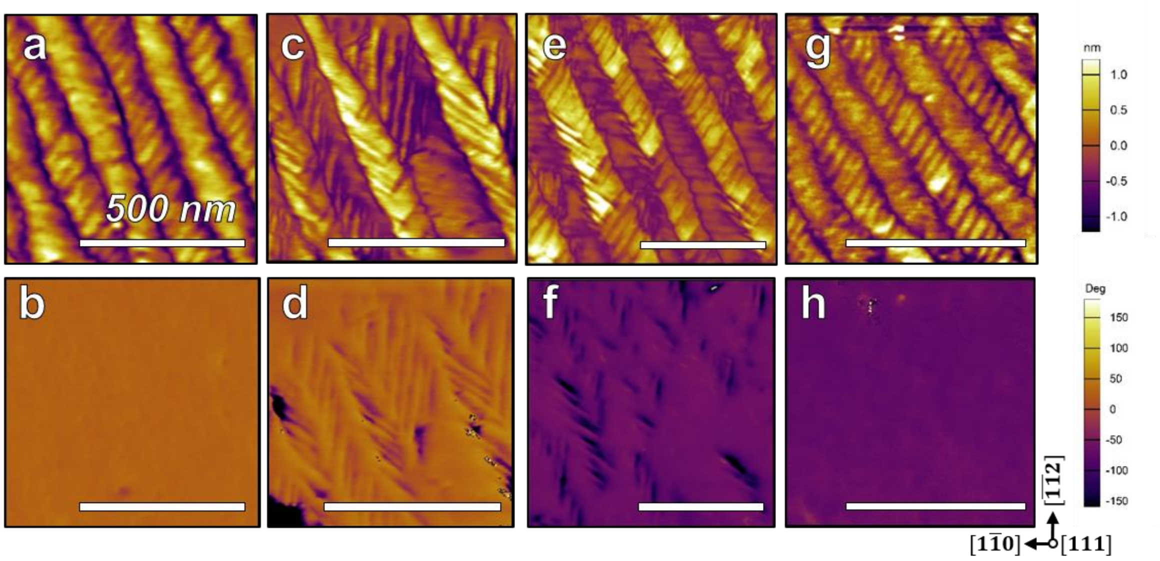

Supplementary Figure 9 | Piezoresponse force microscopy characterization of domain structures. Lateral amplitude (**a**, **c**, **e**, **g**) and vertical phase (**b**, **d**, **f**, **h**) piezoresponse force microscopy images for the **a**, **b**, initial fully-up-poled state, **c**, **d**, intermediate state poled at 1.1 V, **e**, **f**, intermediate state poled at 1.6 V, and **g**, **h**, final fully-down-poled state poled at 3.0 V.

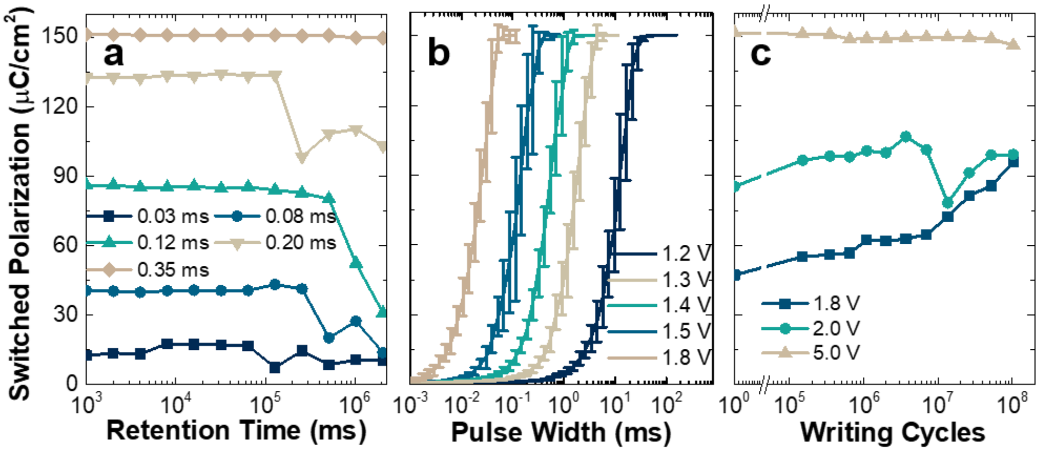

Supplementary Figure 10 | Pulse-switching measurements in (001)-oriented heterostructures. a, Retention measurements probing the time stability of intermediate-polarization states. **b,** Repeatability measurements in wherein the error bars were generated after repeating each pulse-switching measurement 10 times. **c**, Endurance measurements of the switched polarization as a function of writing cycles.

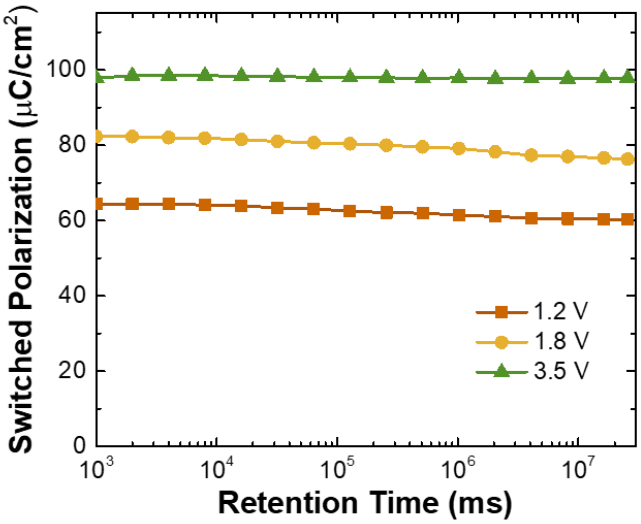

Supplementary Figure 11 | Retention measurement in (111)-oriented heterostructures. Retention measurements probing the time stability of various intermediate-polarization states in (111)-oriented heterostructures up to 2×10^7 ms (7.5 hours).
